# Formal Thought Disorders–Historical Roots

**DOI:** 10.3389/fpsyt.2018.00572

**Published:** 2018-11-09

**Authors:** Joana Jerónimo, Tiago Queirós, Elie Cheniaux, Diogo Telles-Correia

**Affiliations:** ^1^Faculdade de Medicina da Universidade de Lisboa, Lisbon, Portugal; ^2^Hospital de Santa Maria, Centro Hospitalar Lisboa Norte, Lisbon, Portugal; ^3^Faculdade de Ciências Médicas da Universidade do Estado do Rio de Janeiro, Rio de Janeiro, Brazil; ^4^Instituto de Psiquiatria da Universidade Federal do Rio de Janeiro, Rio de Janeiro, Brazil

**Keywords:** psychopathology, descriptive psychopathology, thought disorders, formal thought disorders, schizophrenia, history of psychiatry

## Abstract

In this article the authors intend to review in an intelligible and comprehensive way the historical roots of Formal Thought Disorders. Early descriptions of thought disorders date back to the XIX century with Esquirol, but it was in the first half of the XX century that several authors introduced the main features of the actual concept of Formal Thought Disorders. Emil Kraepelin described *akataphasia* (inability to find the appropriate expression for a thought) in patients with *dementia praecox* (a term that some years later was replaced by schizophrenia). Bleuler and Kretschmer also identified in schizophrenic patients a generalized “loosening of associations” and Carl Schneider described several Formal Thought Disorders such as derailment, fusion, omission, suspension and driveling. At the end of the XX century Nancy Andreasen studied the classical descriptions regarding Formal Thought Disorders, reclassified them and also introduced a scale to assess them. Although the specificity of these symptoms in schizophrenia and psychosis has been a source of controversy among the different authors, the importance given to their presence in these mental disorders is universal. We defend that it is crucial that these historical and conceptual elements are grasped in order to assess Formal Thought Disorders for clinical and research purposes.

## Introduction

The term psychopathology derives from two Greek words: “psyche” meaning “soul,” and “pathos” meaning “suffering.” Throughout the evolution of the term, it has been used under two strands: explanatory psychopathology and descriptive psychopathology (DP). While the former includes explanations of symptoms based on specific lines of thought (e.g., psychodynamic, cognitive-behavioral, neuroscientific or biological), the later refers only to the precise description and categorization of psychopathological manifestations (1).

According to Berrios, DP can be defined as a systematic set of general principles, terms and rules of application, used to capture and describe aspects of behavior that are assumed to result from a psychic or organic dysfunction (1).

The DP, as it is today, results from a combination of historical contributions from several centuries of clinical research in psychiatry.

Different kinds of classification of psychopathological symptoms were proposed to guide the development of assessment scales for clinical and research purposes and facilitate peer communication.

As Jaspers stressed, it is impossible to order and classify satisfactorily the phenomenological findings, at least for now, but we must sort the phenomena somehow provisionally and “this is best done by a classification which gives some plastic impression of what the facts will naturally yield” [(2), p60]. He suggested the organization of symptoms into 8 groups: (1) awareness of the objects (anomalies of perception), (2) experience of space and time, (3) awareness of the body, (4) delusion and awareness of reality, (5) feelings and affective states, (6) urge, drive and will, (7) awareness of the self, (8) phenomena of self-reflection (2).

It was based on Jaspers' classification that Fish, in his book *Clinical Psychopathology: Signs and Symptoms in Psychiatry*, presented a psychopathological classification that still guides the classification of psychiatric symptoms in many countries. Fish' classification is based on several categories: (1) disorders of perception, (2) disorders of thought and speech, (3) disorders of memory, (4) disorders of emotion, (5) disorders of the experience of self, (6) disorders of consciousness and (7) motor disorders (3).

According to Fish, thought disorders can be generically divided into (1) disorders of the stream of thought, (2) disorders of the possession of thought, (3) disorders of the content of thinking, (4) disorders of the form of thinking. In formal thought disorders (FTD) the organization and associative process of thinking, mainly the abstract component and conceptualization, are impaired. On the other hand, in content thought disorders the disturbance lies on the content of the patients' thought (e.g., delusions) (3).

Initially this psychopathological category has been introduced as almost specific to schizophrenia. However, it is now accepted that this symptom may also present itself in other situations such as organic cerebral disorders (e.g., in confusional states) and in other psychiatric disorders, such as depression and mania (4).

This article intends to review the evolution of the concept of formal thought disorders (FTD) taking into account the contributions of the various authors who have studied the subject throughout the history of psychiatry.

## FTD in the XIX century

Esquirol (5) was the first author to hint at the presence of a primary pathology of the faculty that is in charge of coordinating ideas (5).

Some years later, Prichard uses the term “incoherence” for the situations where there are flaws in the connection between thoughts, which often arise in psychiatric patients (6).

Guislain (7) also uses the term “*incoherence des ideées*” and proposes a distinction between thought (including FTD) and speech disorders (7).

In 1867, Griesinger distinguished for the first time the “formal deviations” (*formale Abweichungen*) from “false contents” (*falscher Inhalt der Gedanken*) (8), nowadays known as formal and content thought disorders, respectively.

The disorders of thought were also reported by Hecker in 1871, who wrote about a peculiar departure from normal logical sentence structure, with frequent changes in direction that may or may not lose the train of thought (9).

Jules Séglas (10) gave an important contribute to the development of thought disorders (including FTD) (10). According to him, all the symptoms with diagnostic value for mental disorders were expressed through language and gesture. In turn, changes in speech could be divided into *dyslogies* (thought disorders), *dysphasies* (language disorders), or *dyslalies* (speech disorders). This author described four types of *dyslogies*: *Tempo* (increased or decreased rate of thought), *Form* (changes in plaintiveness of thought, verbigeration, etc), *Syntax* (e.g., referring to the self in the third person or disintegration of sentence construction), *Content* (including fixation in certain themes, stereotypes and neologisms) (6, 10).

## First half of the XX century

In the beginning of the twentieth century, Renée Masselon (1902) included in the chapter of *Psychologie des Déments Précoces* the description of some symptoms compatible with the actual definition of FTD that he classified as “language disorders” (11).

In 1910, Emil Kraepelin introduces the term *akataphasia* as one of the linguistic expression disorders frequent in *dementia praecox*. In this case the patients either do not find the appropriate expression to their thoughts and produce words with similar sounds, or they let their speech follow in a totally different direction (12). These changes, which are closely related to the phenomena described by other authors in formal thought disorders, are presented by Kraepelin in the language disorders section.

The difficulty in differentiating thought disorders from language and speech disorders (patent in Masselon, Kraeplin, and several other authors) is very common throughout the history of psychopathology. As already explained by Jaspers, it is related to artificiality and subjectiviy, both present in psychopathology classifications.

In 1914, Kleist described that some patients used words idiosyncratically to cover a greater range of meaning than they mentally encompass. He called these *stock* words or phrases. This psychopathological disorder reflected a poverty of words and syntax and also an active tendency for words to intrude into thoughts, and therefore speech. According to this author, in schizophrenic patients, the constellation of associations between words is also disordered and they often presented apparently irrelevant associations, even though they seem appropriate subjectively to the patient himself (13).

Bleuler (14) studied this subject in much more detail than any author before. He described FTD as a direct consequence of the “*loosening of association”* and a fundamental disturbance in schizophrenia. In his opinion, there would be an inability to associate ideas due to the absence of a central deterministic idea. Thoughts arise linked to each other by means of idiosyncratic causal connections, leading to a production of distorted concepts characterized by condensation, displacement, and symbolism. This way patients present thoughts that are disconnected from reality (autistic) (14).

Kretschmer, similarly to Bleuler also regarded FTD in schizophrenia as a result of a generalized “loosening of association” in mental functions (15). Babcock (1933) also described FTD in the schizophrenic patients but he stressed that they resulted from a slowing of all intellectual processes and not from the “loosening of association” that other authors suggested (16).

The work of Carl Schneider (1930) was also a key contribution in the history of FTD. He described a number of changes in thought that could be regarded as FTD: derailment, fusion, suspension and drivelling (16). According to Schneider, the three components of normal thinking (constancy, organization, and continuity) are disturbed in schizophrenic thinking. *Derailment* (*entgleisen*) consists in the breakdown in association so that the main thought flows into another subsidiary unrelated thought (e.g., ”I'm going to take the bus, I go to my parents' house, the president controls my ideas, the cameras are in my room”). In *fusion* (*verschmelzung*) there is some preservation of the normal chain of associations, with juxtaposition of heterogeneous and incomprehensible contents. In other words, several ideas A, B, C are interconnected. (e.g., “I know that the martians have been chasing me since that day on the beach. The shape of my room has changed since I have these supernatural powers and my mother knows it, so the martians will come back to get me and that beach remains blue, but the powers that I have my mother never denied them”). *Suspension* consists in the sudden interruption of a certain thought (e.g., ”I am going to take the bus to…today I had lunch and it was fine”). This last phenomenon is very similar to the “block of thought,” a disorder of the stream of thought (3). In *drivelling* there is a miscellany of fragments of heterogeneous thoughts, with loss of associations and loss of sense (3). This can occur when there is a high degree of *derailment* and *fusion*, with or without maintenance of the syntactic structure (17).

Goldstein (18) described a special form of concrete thinking that was present in patients with schizophrenia. This *concrete thinking* or abstraction deficit refers to the inability to make the distinction between the symbolic and the concrete, and also to the incapacity to treat internal and external stimuli conceptually and to delimit them in relation to the surrounding environment. The patient is not able to deal with his experiences conceptually, does not perceive the objects as belonging to a class or category and is incapable of understanding abstraction (19).

Norman Cameron (20) emphasized the lack of connections between successive thoughts that could be present in psychiatric patients. He termed this phenomenon as *asyndesis*. Cameron also includes the following as FTD: *over-inclusiveness* when the patient cannot maintain the boundaries of a concept (including in it attributes from other concepts, e.g., the patient may confuse “living room” with “living room chair”), *metonyms* that mean the use of imprecise expressions in which a term of a phrase is used instead of more accurate ones (e.g., “I'm going to eat a plate”), *interpenetration of themes* (difficult to differentiate from C. Schneider's fusion concept) and *thought fragmentation* (bearing many resemblances to C. Schneider's derailment concept) (20).

Hamilton (19), in his main work “*Die beginnende Schizophrenie*,” describes in *apophany* several psychopathological phenomena compatible with FTD, such as fusion and drivelling (original concepts from C. Shneider) and alogy (thought without logic) (21).

## End of the XX century/beginning of the XXI century

Frank Fish (3) has brought together the classic descriptions of psychiatric symptoms, and based on them he presented a psychopathological classification. It included the FTD, which he described and organized according to several classical authors. Fish also subdivided them into negative or positive: while in the negative FTD the patient loses his capacity to think (even though he doesn't produce abnormal concepts), in the positive FTD the patient produces false concepts resulting from the fusion of several disconnected elements. After Fish' death, the text was revised and updated by Max Hamilton in 1974 and 1985 (19).

Other authors have developed concepts that are very close to the original meaning of FTD. Among them, Arieti points out, in 1969, that while the process of human brain evolution has shown continual rise from the concrete to the abstract, in schizophrenia concrete forms of thought re-emerge. Therefore, not only schizophrenic patients, but also little children tend to show a paleological logic that is progressively replaced by the Aristotelian logic of adults, using second-order cognitive processes compared to those used by normal subjects. In an example cited by Arieti, a schizophrenic patient says she is the Madonna. The paleological reasoning behind this statement can be interpreted thusly. E.g., the patient thinks: the Madonna is a virgin. I am also a virgin, so I am the Madonna. Arieti defines this idea as paleological thought, which is applied by the patient to understand psychic events that are complex and do not respond to a logical linearity (22). The schizophrenic patient abandons Aristotelian logic and adopts paleological logic to escape anguish, because, according to Aristotelian logic reality is interpreted as threatening and unbearable (23). In conclusion, Arieti argues that paleological thought expresses a less integrated and evolved mode of thought (22).

Nancy Andreasen (4) argues that the set of psychiatric symptoms gathered under the name of FTD should be redefined and regrouped into new categories of thought, language and communication disturbances (4). This author criticizes some aspects about the way that FTD have been addressed over the time. Among these, she points out that it is not correct to deal with FTD as if they represented a unitary dimension, when in reality all of these symptoms are conceptually divergent. Another aspect with which Andreasen does not agree is that FTD are traditionally considered to be specific to schizophrenia, and she stresses that it has been concluded in several studies that not only these symptoms may appear in other psychiatric or medical diseases (or even in healthy individuals) but also they aren't present in many patients with schizophrenia.

Therefore, Andreasen created a scale in which the classic FTD are subdivided in three groups (24–26): (1) Communication disturbances—when the speaker does not meet the necessary requirements for the listener's understanding (poverty of content of speech, pressure of speech, distractible speech, tangentiality, derailment, stilted speech, echolalia, self-reference, circumstantiality, loss of goal, perseveration, and blocking); (2) Language disturbances—when the speaker violates the semantic and syntactic conventions (incoherence, clang association (e.g., assonance), neologisms, use of word approximations); (3) Thought disturbances—when only thinking alone seems affected (poverty of thought and illogicality aberrant inferential processes). According to this classification FTD should rather be referred as disorders of thought-language-communication (24). Andreasen demonstrated a good reliability for this classification system and also demonstrated that these psychopathological findings were not specific to schizophrenia and were also common in other mental disorders (e.g., Mania) (27).

Sims, in the first edition of his book, from 1988, defends the use of the expression “disorder of the thinking process” instead of FTD. According to him, abnormalities of thinking process “cannot be easily related to any clearly described, already established notion of what normal processes are”[(28), p129].

With the release of Diagnostic and Statistics Manual IV (DSM-IV) in 1994, the term “disorganized speech” was chosen instead of the classical FTD designation: “ Because of the difficulty inherent in developing an objective definition of ”thought disorder,“ and because in a clinical setting inferences about thought are based primarily on the individual's speech” [(29), p276]. It is added in this manual that these symptoms are not specific of schizophrenia.

In the fifth edition of DSM (DSM-5), published in 2013, it was decided that this designation should remain (30).

A lack of consensus regarding a better way to conceptualize and assess these symptoms has remained so far. This situation could be a case for concern since psychiatric research (clinical and neurobiological) should ideally be grounded in unambiguous descriptive psychopathology (30).

## Discussion and conclusions

Since Esquirol, there have been reports of certain psychopathological disorders in which the main characteristic is the failure of association between successive thoughts. In the XIX century Griesinger distinguished for the first time the “formal deviations” from “false contents” among thought disorders. But it was in the first half of the twentieth century that the greatest investment was given to this concept. Although several authors such as Kraepelin, Masselo and Kleist had contributed to this cause, Bleuler was the one who invested the most, not only in the description, but also concerning the etiopathogenic basis of the FTD. Other authors such as Carl Schneider, Kurt Goldstein and Norman Cameron have made an essential contribution to the psychopathological semiology of FTD. At the end of the XX century, there was an attempt to bring together and reformulate the historical contributions of the former authors on this subject, notably with Nancy Andreassen.

As we have seen, several concepts related to FTD were created by the great authors of psychiatry and it seems difficult to gather all within the same theoretical model. Phenomenological psychopathology is characterized by a lack of uniformity in relation to its terms and concepts. Thus, it is possible that the same phenomenon has been designated in different terms by different authors.

Accordingly, for example, terms such as “incoherence” by Prichard, “loosening of association” by Bleuler and “derailment” by Carl Schneider, may all represent the same concepts in FTD.

Although the specificity of these symptoms in schizophrenia and psychosis has been a source of controversy among the different authors, the importance given to their presence in these mental disorders is consensual. It was demonstrated in a recent systematic review (which included 120 articles, based on several ways of defining and assessing FTD) that FTD are a common symptom of psychosis and may be considered a marker of illness severity (31).

In recent times there has been a reflection on what are the most important symptoms in schizophrenia and which should base the translational neuroscience research in this area (32). There seems to be no doubt about the importance of FTD in schizophrenia and thus these symptoms seem to be a good candidate to guide research that seeks to find the neurobiological correlates of schizophrenia. Some evidence has already been found in this area, such as several structural and functional changes in the lateral temporal lobes that have been related to FTD (again based on several ways of defining and assessing FTD) (33).

Crow (34) defended the idea that FTD could be derived from an absence of hemispheric asymmetry in language areas. His work supports genetic association between language and schizophrenia, defending that the genetic mutation that allowed the emergence of language in humans, can be responsible for their vulnerability to failures, which may be clinically manifested as schizophrenia (34, 35).

One of the major problems associated with FTD, and psychopathology in general, is the lack of uniformity of concepts and ways of accessing symptoms. As well as there have been several ways of defining FTD, also multiple methods of assessing this symptom have been used.

General psychopathological scales such as the Scale for the Assessment of Positive Symptoms [SAPS] (26), the Scale for the Assessment of Negative Symptoms [SANS] (27), the Positive and Negative Syndrome Scale [PANSS] for schizophrenia (36), the Brief Psychiatric Rating Scale [BPRS] (37), have some items dedicated to FTD. Some specific FTD scales have also been developed such as the Thought and Language Disorder (TALD) scale (38), Thought, Language, and Communication Disorders (TLC) scale (25), The Thought and Language Index (TLI) (39), The Thought Disorder Index (TDI) (40).

This variability in the ways of defining and measuring this psychopathological disorder has important consequences in both clinical and translational research.

In this article we intended to describe in an intelligible and comprehensive way the historical roots of the FTD concept. We defend that it is crucial that these elements are grasped in order to assess FTD for clinical (eg. schizophrenia and psychosis diagnosis) and research (clinical and translational) purposes.

As Andreasen (40) pointed out: “Applying technology without companionship of wise clinicians with specific expertise in psychopathology will be a lonely, sterile and perhaps fruitless enterprise.” (41).

## Author contributions

DT-C conceived and directed the work. JJ took the lead in writing the manuscript. JJ and TQ wrote the manuscript with input from all authors. All authors provided critical feedback.

### Conflict of interest statement

The authors declare that the research was conducted in the absence of any commercial or financial relationships that could be construed as a potential conflict of interest.
